# Identification of Neonatal Factors Predicting Pre-Discharge Mortality in Extremely Preterm or Extremely Low Birth Weight Infants: A Historical Cohort Study

**DOI:** 10.3390/children11121453

**Published:** 2024-11-28

**Authors:** Zhenyuan Dai, Xiaobing Zhong, Qian Chen, Yuming Chen, Sinian Pan, Huiqing Ye, Xinyi Tang

**Affiliations:** 1 Department of Pediatrics, The Third Affiliated Hospital, Sun Yat-sen University, Guangzhou 510630, China; daizhy3@mail.sysu.edu.cn (Z.D.); zhongxb5@mail.sysu.edu.cn (X.Z.); chenq563@mail.sysu.edu.cn (Q.C.); pansn@mail.sysu.edu.cn (S.P.); yhuiq@mail.sysu.edu.cn (H.Y.); 2 Department of Epidemiology, School of Public Health, Sun Yat-sen University, Guangzhou 510080, China; chenyum@mail.sysu.edu.cn

**Keywords:** extremely preterm infants, extremely low birth weight infants, mortality, predictive factors, FiO_2_, surfactant, umbilical vein catheterization

## Abstract

Background/Objectives: This study identified early neonatal factors predicting pre-discharge mortality among extremely preterm infants (EPIs) or extremely low birth weight infants (ELBWIs) in China, where data are scarce. Methods: We conducted a retrospective analysis of 211 (92 deaths) neonates born <28 weeks of gestation or with a birth weight <1000 g, admitted to University Affiliated Hospitals from 2013 to 2024 in Guangzhou, China. Data on 26 neonatal factors before the first 24 h of life and pre-discharge mortality were collected. LASSO–Cox regression was employed to screen predictive factors, followed by stepwise Cox regression to develop the final mortality prediction model. The model’s performance was evaluated using the area under the curve (AUC) of the receiver operating characteristic, calibration curves, and decision curve analysis. Results: The LASSO–Cox model identified 13 predictors that showed strong predictive accuracy (AUC: 0.806/0.864 in the training/validation sets), with sensitivity and specificity rates above 70%. Among them, six predictors remained significant in the final stepwise Cox model and generated similar predictive accuracy (AUC: 0.830; 95% CI: 0.775–0.885). Besides the well-established predictors (e.g., gestational age, 5 min Apgar scores, and multiplicity), this study highlights the predictive value of the maximum FiO_2_. It emphasizes the significance of the early use of additional doses of surfactant and umbilical vein catheterization (UVC) in reducing mortality. Conclusions: We identified six significant predictors for pre-discharge mortality. The findings highlighted the modifiable factors (FiO_2_, surfactant, and UVC) as crucial neonatal factors for predicting mortality risk in EPIs or ELBWIs, and offer valuable guidance for early clinical management.

## 1. Introduction

With the rapid advancement of perinatal medicine and neonatology in recent years, the management and outcomes of extremely preterm infants (EPIs, <28 weeks of gestational age [GA]) and extremely low birth weight infants (ELBWIs, <1000 g at birth) have markedly improved worldwide, resulting in a notable decline in mortality rates [1]. However, due to the inherent challenges of developmental immaturity and disparities in healthcare quality, EPIs and ELBWIs continue to be leading causes of death among perinatal and under-five children [2].

Mortality rates for EPIs vary significantly across regions with different economic and healthcare conditions [3,4,5,6,7]. In developed countries, the mortality rate for infants born at or below 28 weeks of gestation has fallen to below 25%. In the United States, from 2013 to 2018, the pre-discharge mortality rate among 10,877 EPIs born at 22–28 weeks of gestation was 21.7% [3]. An international comparison of survival rates for preterm infants born at 24–29 weeks of gestation across neonatal networks in 10 countries/regions revealed that Japan had the highest survival rate at 93.3%, while Spain had the lowest at 78.1% between 2007 and 2013 [5]. A study including 8514 neonates born before 28 weeks across 68 tertiary hospitals reported a much higher mortality of 37.7% in China from 2010 to 2019 [6]. These findings highlighted considerable variability in EPI mortality rates across different hospitals, suggesting significant potential for improvement, particularly in less-equipped facilities. Identifying early predictive and modifiable factors is crucial to inform clinical decision-making and improve care outcomes.

Multiple studies have explored the risk factors associated with pre-discharge mortality in EPIs and ELBWIs [8,9,10]. Key risk factors identified include extremely low GA or birth weight (BW) [8], infections, early-onset respiratory distress syndrome (RDS) [11], maternal health conditions (such as hypertension and diabetes), and pregnancy complications (such as preeclampsia and placental abruption) [10]. The use of antenatal corticosteroids and enhancements in neonatal intensive care have been shown to significantly reduce mortality and the incidence of severe complications in EPIs [11,12].

In China, a limited number of studies have examined these risk factors. For instance, Lin et al. reported an increased mortality risk among infants with a BW < 750 g or a GA < 28 weeks, but a decreased risk for small for gestational age (SGA) infants in 250 ELBWI infants from national multi-centers [7]. Similarly, Zhu et al. found that GA, BW, premature rupture of membranes (PROM), and antenatal steroid use were associated with improved survival. In contrast, being SGA, male, having multiple births, low Apgar score, or having maternal diabetes were linked to poorer survival outcomes in ELBWIs across 31 provinces in China [6]. Despite these findings, variations in medical care across different regions and hospitals, and differences in the factors studied, have resulted in substantial discrepancies in identified predictors and risk factors. Additionally, limited data are available in China, with significant heterogeneity in the quality of medical care across developed regions.

This study aims to investigate, from the perspective of neonatologists, the predictive and risk factors of pre-discharge mortality based on the basic characteristics, laboratory findings, and clinical management of newborns on their first day of life. The goal is to provide valuable insights for early mortality assessment, prediction, and potential intervention measures in EPIs or ELBWIs.

## 2. Materials and Methods

### 2.1. Study Subjects

The study subjects included in this historical cohort study were selected from patients born and hospitalized at the Third Affiliated Hospital of Sun Yat-sen University (Guangzhou, South China) between 2013 and 2024. From 28,565 neonatal inpatient registration records, we identified 702 neonates with a BW < 1.50 kg. After reviewing detailed electronic medical records, those meeting the inclusion criteria (GA < 28 weeks or BW < 1.0 kg) were selected. We excluded cases where guardians voluntarily abandoned treatment for non-medical reasons, outcomes were unknown due to transfer to other hospitals, survival outcomes were unclear due to discharge before reaching the required discharge weight (≥2.0 kg), and neonates were not born at our hospital. Moreover, we excluded stillbirths, delivery room deaths, and cases where parents opted for palliative care and did not admit the infant to the neonatal intensive care unit. Finally, a total of 211 cases were included in this study.

### 2.2. Data Collection

Data were collected by reviewing electronic medical records, with two independent reviewers extracting the following information: basic characteristics at birth (sex, length, weight, GA, mode of delivery, SGA), maternal factors (age, antenatal steroids), blood test indices within 2 h after birth (base excess [BE], lactate, pH, white blood cell count [WBC], hemoglobin [HGB], and C-reactive protein [CRP]), vital signs (Apgar scores at 1 [Apg-1], 5 [Apg-5], and 10 [Apg-10] minutes, body temperature, asphyxia, heart rate, systolic blood pressure, diastolic blood pressure, and mean arterial pressure at admission within 30 min), clinical interventions within 24 h post-birth (use of surfactant, invasive mechanical ventilation, maximum fraction of inspired oxygen [FiO_2_], use of umbilical venous catheterization [UVC]) or peripherally inserted central catheters [PICCs], clinical interventions after 24 h (use and duration of antibiotics, days of mechanical ventilation, days of UVC, time to full enteral feeding, breastfeeding), and major complications during hospitalization (pulmonary hemorrhage, sepsis, edema, intracranial hemorrhage, RDS, bronchopulmonary dysplasia [BPD], patent ductus arteriosus [PDA], cholestasis, necrotizing enterocolitis [NEC], arrhythmia, and weekly weight measurements). The maximum appropriate FiO_2_ was defined as the highest oxygen concentration required during mechanical ventilation to achieve and maintain transcutaneous oxygen saturation (SpO_2_) ≥ 95%. UVC placement is recommended as a non-invasive procedure immediately after birth. In cases where UVCs were unable to be placed primarily due to technical reasons, peripheral venous cannulation was performed and followed by PICC insertion within 1–3 days once the neonate’s condition stabilized. We collected data on clinical interventions after 24 h and major complications during hospitalization for completeness. However, to avoid potential reverse causation, only data from the first 24 h of life were included in the predictive analysis.

### 2.3. Data Analysis

#### 2.3.1. Data Processing

Missing values in continuous variables were imputed using the mean of five multiple imputations if the missing rate was within 10%. C-reactive protein (CRP) was excluded from the analysis due to a missing rate exceeding 10%. Outliers in continuous variables, defined as values exceeding the mean ± 3 standard deviations (SD), were replaced with the nearest values. A total of 26 predictive variables were included in the analysis, excluding survival days and mortality events.

#### 2.3.2. Data Description and Univariate Analysis

The essential characteristics of the study subjects were described and compared between the survival and mortality groups. Continuous variables that followed a normal distribution and had homogeneity of variance were described using mean ± SD, and differences between groups were analyzed using *t*-tests. For non-normally distributed variables or those with unequal variances, the median and interquartile range (IQR) were used, and group differences were analyzed using the Wilcoxon rank-sum test. Categorical variables were described as frequencies (percentages) and analyzed for group differences using the chi-squared test. Hazard ratios (HR) and 95% confidence intervals (CI) for the categorical variables were estimated using the univariate Cox regression model.

#### 2.3.3. Screening Predictive Features Using a LASSO–Cox Model

Predictive features were screened using the LASSO (least absolute shrinkage and selection operator) method to fit a Cox proportional hazards regression model [13]. Survival time and death were used as the dependent variables, with all 26 characteristics as predictors. The 211 neonatal cases were randomly divided into training and validation sets in a 65:35 ratio.

The LASSO method, implemented via the “*glmnet*” package (version 4.1-8) in R, was used to determine the impact of different penalization strengths (lambda values) on the regression coefficients [14]. A coefficient path plot was generated to visualize how the coefficients changed as lambda varied. To fit the Cox regression model, the “*cv.glmnet*” function was applied, incorporating 20-fold cross-validation to optimize model parameters and evaluate stability and performance across subsets of the data [14].

Additionally, five different gamma values (ranging from 0 to 1) were tested using deviance as the performance metric. The optimal lambda value, corresponding to the point of minimum deviance, indicated the best trade-off between model fit and generalizability. The predictive performance of the LASSO–Cox model was evaluated in both the training and validation sets using the concordance index (C-index) and the area under the curve (AUC) of the receiver operating characteristic (ROC) curve [14].

#### 2.3.4. Identification of Final Predictive Features Using a Stepwise Cox Model

Predictive factors with coefficients that did not shrink to zero at the minimum lambda in the LASSO–Cox model were further evaluated using a backward stepwise multivariate Cox regression analysis. This approach identified significant independent features that were retained in the final model across all subjects. Based on this final model, the following analyses were then conducted: (1) HRs and 95% CIs were calculated to quantify the associations between predictive features and mortality risk; (2) a linear risk score for mortality was calculated for each infant, with infants categorized into High (>median) and Low (≤median) risk groups based on the median risk score, and survival curves for these groups generated using the “*survival*” package (version 3.5-8) in R [15]; (3) a nomogram was constructed with the “*nomogram*” function from the “*rms*” package (version 6.6-0) [16] to predict mortality risks at 2, 9, and 50 days, corresponding to the median, 75th percentile, and 100% survival times in the mortality group; (4) the model’s performance was evaluated using the ROC-AUC and a calibration curve generated with the “*calibrate*“ function from the “*rms*” package [16]; (5) and clinical utility was evaluated using DCA at different risk thresholds using the “*ggDCA*” package (version 1.2) [17,18].

The proportional hazards assumption for each variable in the final model was tested using the “*cox.zph*” function from the “*survival*” package in R [15]. All *p*-values were greater than 0.1, indicating that the proportional hazards assumption was adequately satisfied. Collinearity among the variables in the final model was assessed using the variance inflation factor (VIF) [15]. All variables exhibited VIF values well below 10 (<2), suggesting minimal collinearity issues [15].

All statistical analyses were conducted using R software (version 4.4.0) in RStudio (2024.04.1 Build 748, © 2009–2024 Posit Software, PBC, http://www.r-project.org/ accessed on 20 July 2024). A two-tailed *p*-value < 0.05 was considered statistically significant.

## 3. Results

### 3.1. Comparison of Basic Characteristics of Newborns by Survival Status

From over 20,000 newborns born and hospitalized in our institution, we identified 211 neonates with definite outcomes, either with a GA of <28 weeks or a BW of <1000 g. Of these, 92 (43.6%) died. We compared basic characteristics and clinical interventions within 24 h of birth. The results, presented in Table 1 and Table 2, show that the median observation time for survivors was 67 days (IQR: 56–78), while the median observation time for the deceased group was 2 days (IQR: 1–9).

Significant differences were observed between the deceased and surviving groups in several neonatal parameters in the univariate analysis. The values or proportions of GA at birth, BW, Apgar scores (1, 5, and 10 min), base excess (BE), pH, body temperature, year at birth, use of antenatal steroids, cesarean delivery, and immediate postnatal UVC were significantly higher in the surviving group compared to the deceased group (all *p* < 0.05). Conversely, higher values or proportion of blood lactate, maximum FiO_2_, maternal age, twin or multiple births, perinatal asphyxia, and use of mechanical ventilation were found in the deceased group compared to the surviving group (all *p* < 0.05).

Other variables, including sex, white blood cell count, hemoglobin levels, the use of surfactant, heart rate, systolic and diastolic blood pressure, mean arterial pressure, and SGA status, did not differ significantly between the two groups (*p* > 0.05).

### 3.2. LASSO–Cox Regression for Selecting Important Features in Neonatal Mortality

**Figure 1:**
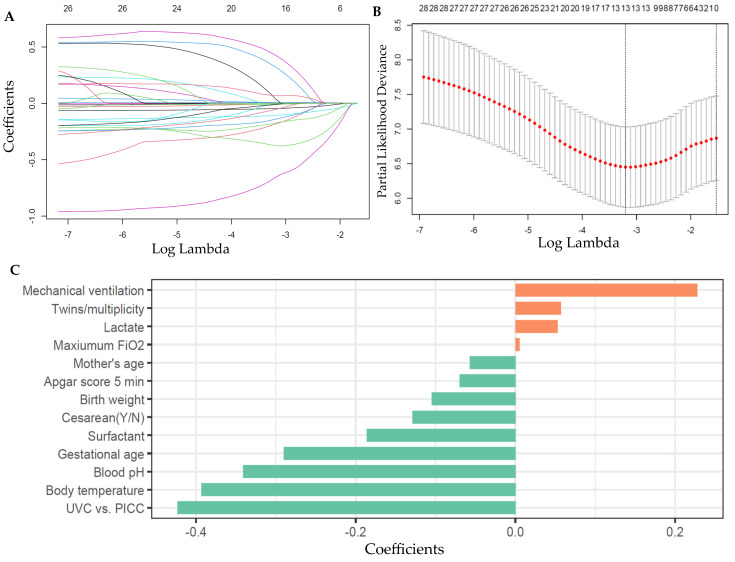
Predictor selection and their coefficients for pre-discharge deaths from the LASSO–Cox regression model with 20-fold cross-validation in the training set. (**A**) LASSO regression variable selection dynamic plot. Each line corresponds to a predictive variable and its coefficient (**B**) Cross-validation plot for LASSO regression model. A total of 13 variables with nonzero coefficients were selected by deriving the minimum lambda. (**C**) Coefficients of the selected predictors for deaths from the LASSO–Cox regression model. LASSO, least absolute shrinkage and selection operator; PICC, peripherally inserted central catheter; UVC, umbilical vein catheterization.

Using LASSO–Cox regression, 13 variables with non-zero coefficients were identified at the “*lambda.min*” value (Figure 1). Factors positively associated with mortality included mechanical ventilation, twins/multiplicity, blood lactate, and maximum appropriate FiO_2_. Conversely, factors negatively correlated with mortality, ranked by importance, included UVC (compared to PICC), body temperature, blood pH, GA, surfactant doses, cesarean delivery, BW, Apg-5, and maternal age.

**Figure 2:**
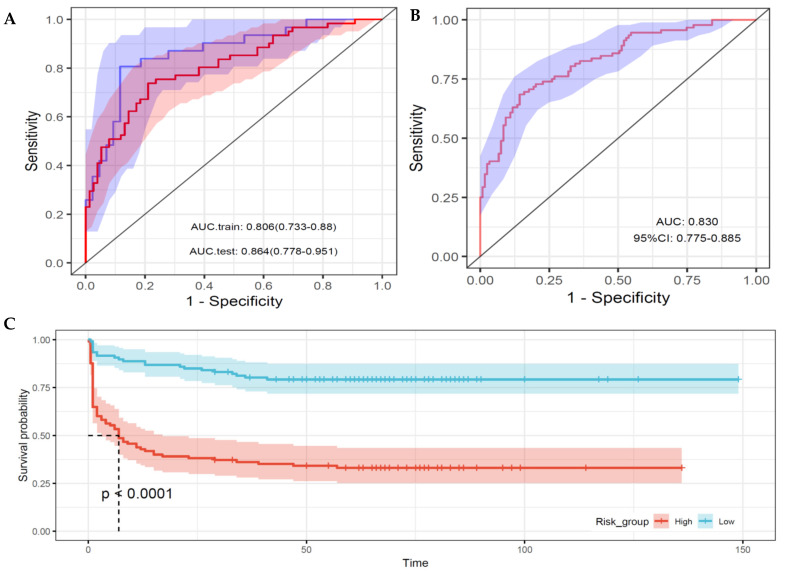
Performance of the predictive models for pre-discharge mortality. (**A**) Receiver operating characteristic curve (ROC) for the LASSO–Cox regression model in the training set (red line) and testing set (blue line), based on a 65:35 split ratio. (**B**) ROC for the final stepwise Cox regression model applied to the entire dataset using 13 predictors selected from the LASSO–Cox model. (**C**) Kaplan–Meier survival curves for high- and low-risk groups, stratified by the median risk score predicted from the final stepwise Cox regression model. A significant difference in survival is observed between the two groups at the median survival/observation time (*p* < 0.0001). Shaded areas indicate the 95% confidence intervals for each curve.

The model’s predictive performance, assessed using the C-index, was 0.779 in the training set and 0.772 in the validation set. The ROC-AUCs were 0.806 (95% CI: 0.733–0.880) and 0.864 (95% CI: 0.778–0.951) in the training and validation sets (Figure 2A). At the optimal cutoff values, sensitivity and specificity were 78.9% and 73.8%, respectively, in the training set, and 88.4% and 80.6% in the validation set. There was no statistically significant difference in ROC-AUC between the training and validation sets (*p* = 0.32).

### 3.3. Predictive Cox Model for Neonatal Mortality Causes

From the 13 features identified by the LASSO–Cox regression model (Figure 1), six statistically significant predictors were retained in the final stepwise Cox regression model (Table 3). Key findings include the following: each additional unit of surfactant was associated with a reduced mortality risk by 33% (HR: 0.67, 95% CI: 0.57–0.80); an increase of one point in Apg-5 was correlated to 17% (95% CI: 8–25%) lower risk; every 10% increase in the maximum FiO_2_ during ventilation was associated with a 17% (95% CI: 8–26%) higher mortality risk; using a UVC (vs. PICC) significantly correlated to reduced mortality risks, with a hazard ratio of 0.37 (95% CI: 0.23–0.60); the mortality risk for twins/multiple births was 2.03 (95% CI: 1.21–3.39) times higher than for singletons; and compared to neonates with a GA of less than 26 weeks, the mortality decreased by 67% (HR: 0.33, 95% CI: 0.18–0.60) at 27.0–27.9 weeks and by 71% (HR: 0.29, 95% CI: 0.14–0.59) at 28.0 weeks or more (*p* < 0.001). Similar results were observed in the sex-stratified analyses (Table 3).

The predictive performance of the model, as measured by the ROC-AUC, was 0.830 (95% CI: 0.775–0.885) for the total sample, 0.826 (0.748–0.904) for male, and 0.814 (0.728–0.900) for female infants, closely aligning with the performance of the LASSO–Cox model (Figure 2B). Stratification based on the median risk score predicted by the final model revealed significant differences in survival curves between the high- and low-risk groups (*p* < 0.001) (Figure 2C).

**Figure 3:**
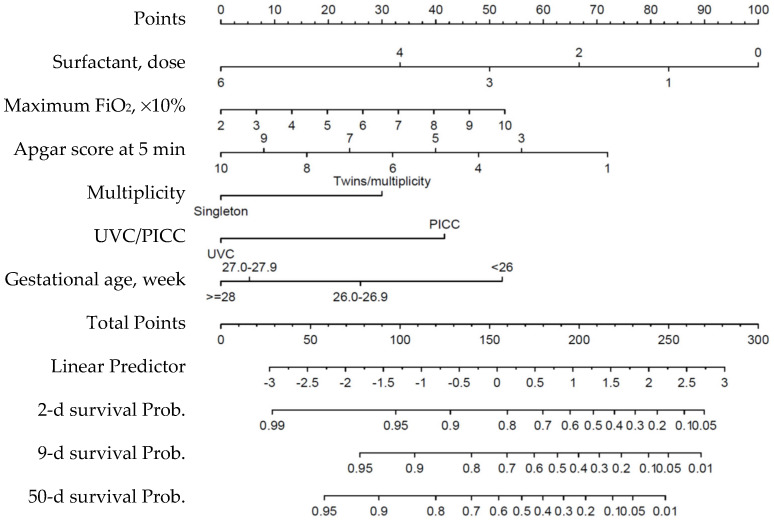
Nomogram for predicting survival probabilities based on the final stepwise Cox regression model. Each predictor corresponds to a specific point value, which is summed to calculate the total points. The total points are then mapped to a linear predictor and survival probabilities at 2, 9, and 50 days, corresponding to the median, 75^th^ percentile, and 100% survival times in the mortality group. PICC, peripherally inserted central catheter; UVC, umbilical venous catheterization.

**Figure 4:**
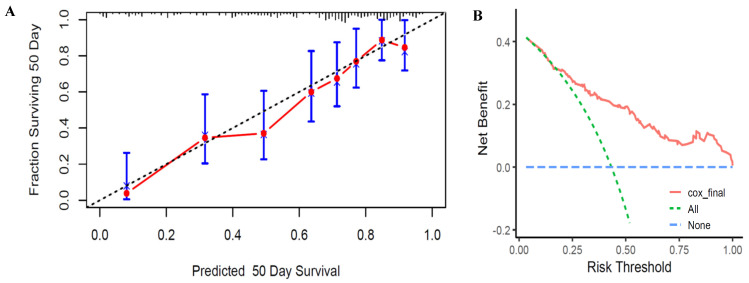
Calibration curves and decision curve analysis of the final stepwise Cox regression model for predicting pre-discharge survival in EPIs or ELBWIs. (**A**) Calibration curves. The *y*-axis represents the actual survival fraction at 50 days (corresponding to the longest survival time observed in the deceased group) after birth, while the *x*-axis represents the predicted 50-day survival probability. The diagonal dotted line indicates perfect prediction by an ideal model, whereas the solid line represents the performance of the final stepwise Cox regression model. (**B**) Decision curve analysis. The red line indicates the net benefit of the Cox regression model across various risk thresholds. The green dashed line represents the “treat-all” strategy, and the blue dashed line represents the “treat-none” strategy.

Based on this final model, a scoring system for the relevant features was developed using a nomogram to predict mortality risks at 2, 9, and 50 days, corresponding to the median, 75th percentile, and 100% survival times in the mortality group, respectively (Figure 3). The calibration curve for the 50-day (pre-discharge) survival prediction exhibited good agreement between predicted and observed outcomes (Figure 4A).

A decision curve analysis was performed to assess the clinical utility of the stepwise Cox regression model at different risk thresholds. The decision curve demonstrated that the model performs well at medium- to high-risk thresholds, providing robust support for clinical decision-making (Figure 4B). This suggests that managing patients using this model may be more appropriate at higher risk levels.

## 4. Discussion

This study analyzed all cases of EPIs or ELBWIs born between 2013 and 2024. We examined 26 neonatal factors on the first day after birth, including general characteristics, laboratory findings, and clinical interventions. We developed a predictive model for pre-discharge mortality using the LASSO–Cox regression method. Further stepwise Cox regression analysis of the factors selected by the LASSO method identified six significant predictors of pre-discharge mortality risk (AUC: 0.830; 95% CI: 0.775–0.885). The final stepwise Cox model showed that using pulmonary surfactant, Apg-5, GA, and UVC use were negatively associated with mortality risk. In contrast, the maximum FiO_2_ required within the first 12 h of life and multiplicity were positively associated with mortality risk.

### 4.1. Comparison of Predictors of Pre-Discharge Mortality in EPIs/ELBWIs

Many prior studies have explored the predictors or risk factors of mortality in EPIs and ELBWIs [8,10,19]. A national prediction model for preterm infants (GA < 32 weeks) in Sweden demonstrated an AUC of 0.858 using 13 perinatal characteristics [10]. Among the most significant predictors, higher Apg-5 and Apg-10, along with higher GA and BW, showed the strongest associations with lower mortality [10]. Additionally, antenatal corticosteroid therapy was significantly linked to lower mortality [10]. A meta-analysis by van Beek et al. identified key predictors of mortality, including size/maturity (GA, BW), birth/delivery variables (Apgar scores, multiplicity, sex), maternal variables (antenatal corticosteroids, race/ethnicity), and respiratory measurements (RDS, FiO_2_) [20]. Another meta-analysis showed that prediction models based on BW and GA alone had AUCs of 0.76 (0.72–0.80) and 0.78 (0.74–0.81), respectively [21]. However, the Clinical Risk Index for Babies (CRIB), a multifactorial model, demonstrated the highest accuracy in predicting pre-discharge mortality, with an AUC of 0.88 (0.86–0.90) [21]. The CRIB model includes BW, GA, congenital malformations, maximal BE, and minimum and maximum appropriate FiO_2_ in the first 12 h [22]. Generally, our findings from univariate or multivariate analysis align with many of these results.

In China, several studies have also investigated the risk factors or predictors of pre-discharge mortality in ELBWIs or EPIs. A national multicenter study involving 250 ELBWIs found that mortality risk was higher among infants with lower BW (<750 vs. ≥750 g) or smaller GA (<28 vs. ≥28 weeks), but lower in SGA infants [7]. Another large study across 31 provinces identified that higher GA and BW, premature rupture of membranes, and antenatal steroid use were associated with decreased mortality. In contrast, lower Apgar-5, being SGA, male, multiple births, or having maternal diabetes were linked to poorer survival outcomes in 8514 EPIs [6]. Two studies in northern China further examined predictors of mortality in EPIs during the early postnatal period. Significant predictors included GA, BW, lactic acid, pH, hypothermia, and antenatal steroid use in one study [23], while another reported associations with BW, chest compressions during delivery, inborn status, severe RDS, invasive mechanical ventilation, and shock [24].

Our study corroborates several key findings in the multivariate model, such as the significance of GA, Apgar-5, and multiple births as predictors of mortality. BW, lactic acid, pH, hypothermia, invasive mechanical ventilation, and antenatal steroid use were consistent with previous findings in univariate analyses. However, no significant associations were found for SGA or sex in our population, possibly due to the relatively small sample size. Notably, our findings underscore the predictive value of maximum appropriate FiO_2_ and highlight the potential clinical benefits of using UVC compared to PICC, as well as administering additional surfactants in the management of EPIs or ELBWIs.

### 4.2. Key Predictors of Pre-Discharge Mortality in This Population

Gestational age: Gestational age (GA) is consistently identified as one of the most critical predictors of neonatal mortality, aligning with findings from previous studies [6,21,23]. Among the 211 infants included in this study, 23 were born at less than 26 weeks, with only 5 (21.7%) surviving. Survival rates increased significantly to 47.7%, 60.2%, and 70.5% for those born at 26.0–26.9 weeks, 27.0–27.9 weeks, and ≥28 weeks, respectively. GA alone is a strong predictor of neonatal mortality, with an AUC of 0.78 (95% CI: 0.74–0.81) [21]. Notably, during the extremely preterm stage (23–26 weeks), survival rates increase rapidly, but the rate of improvement slows beyond 28 weeks [6]. The higher mortality risk associated with lower GA can be attributed to incomplete organ development, increased susceptibility to infections, the vulnerability of the nervous system, and a higher incidence of critical complications related to prematurity. Moreover, differences in medical resources and perinatal interventions greatly influence the relationship between GA and mortality risk [6,25,26].

Use of surfactants: Acute respiratory distress syndrome (RDS) is a critical risk factor for early mortality in EPIs and ELBWIs [27,28]. Acute RDS management aims to maximize survival while minimizing complications such as air leaks and bronchopulmonary dysplasia (BPD). Surfactant therapy has been proven effective in reducing air leaks and mortality rates [11,29]. However, there remains debate regarding the optimal timing and dosing [11,30]. Additionally, the use of surfactants is often limited by their high cost, making it difficult to fully align their administration with clinical needs. Multiple doses of surfactants may reflect the therapeutic requirements for severe RDS but may also lead to better outcomes for RDS cases of similar severity. The multivariate model revealed a strong negative association between the doses of surfactants and mortality in this study. The results suggest that, under similar clinical conditions, multiple adequately dosed surfactant treatments may improve survival outcomes.

Maximum appropriate FiO_2_: RDS management now emphasizes noninvasively clinical assessments, such as work of breathing and FiO_2_, over radiographic diagnosis and grading, and the dynamic monitoring of blood gas analysis. Consequently, FiO_2_ within the first two hours after birth has become an essential predictor of continuous positive airway pressure (CPAP) failure [31]. An increase in maximum appropriate FiO_2_ significantly predicts mortality in EPIs and ELBWIs [32,33], with a 1% increase associated with a 7.5% rise in adverse outcomes risk [32]. Aligning with these observations, our findings showed that every 10% increase in the maximum appropriate FiO_2_ in the first 12 h was associated with an 18% increase in mortality risk (95% CI: 10–27%), underscoring the high predictive value of this simple oxygenation metric, which does not require blood sampling or complex equipment [32].

UVC: UVCs are a commonly used central venous access method for neonates, facilitating quick and reliable vascular access immediately after birth [34,35]. Both EPIs and ELBWIs are eligible for the use of UVC at birth. Compared to PICCs, UVCs allow for non-invasive catheter placement, reducing delays and associated risks [36]. Despite their benefits, the use of UVCs is subject to variability in physician expertise and clinical judgment. Consequently, in this study, only 47.4% of the neonates successfully received UVC, while the remainder were managed with peripheral venous cannulation followed by PICC insertion within 1–3 days once the neonate’s condition stabilized. Previous studies have primarily focused on UVC-related complications, with few reporting its impact on mortality risk. Many studies have demonstrated that extending UVC dwell time significantly reduces the number of painful invasive procedures and radiation exposure, shortens the length of hospital stay [34], and improves critical social competencies [37] without increasing adverse complications [35,38,39,40] compared to PICC placement. Our study found that successful UVC insertion significantly reduced mortality rates, underscoring its importance in clinical management.

Other factors: The Apgar score is a widely used measure of the physical condition of newborns. While the 1 min Apgar score reflects the infant’s immediate condition after birth, the 5 min score is a stronger predictor of neonatal survival, as demonstrated in this study and supported by numerous previous studies [6,10,41]. Consistent with earlier research [6,20,41], our findings indicated that infants from multiple births had a 2.03 times higher mortality risk (95% CI: 1.21–3.39) compared to singletons. Although many studies have reported improved survival outcomes associated with antenatal steroid use [6,20], this trend was only observed in the univariate model in our study. Additionally, we observed better survival outcomes with later years of birth, consistent with findings from other studies conducted in China [6].

### 4.3. Strengths and Limitations

This study reports on the pre-discharge mortality predictors for EPIs or ELBWIs in a Chinese population. Due to the absence of a relevant registry database for mortality predictors of EPIs and ELBWIs in mainland China and the relative difficulty in obtaining raw data, there is currently a lack of related studies in this region. This study is the first to establish a clinical mortality prediction model for EPIs or ELBWIs, providing a valuable reference for understanding mortality predictors in the Chinese population. UVCs and surfactants are highlighted as potentially important early clinical management measures among the predictive factors. The findings of this study offer insights for clinical interventions in EPIs or ELBWIs.

We only selected factors from the first day of life to avoid a reverse causation relationship between predictive factors and survival time, mainly focusing on those within the first 24 h after birth. Factors influencing mortality after the first day, such as pulmonary hemorrhage, infections, antibiotic use, feeding methods, and other clinical measures and complications, were excluded from predictive candidates. However, these factors could also significantly impact pre-discharge mortality later on.

The predictive factors in this study were primarily selected from the perspective of neonatologists, without including sufficient prenatal maternal, family-related factors, or perinatal management measures, which could also influence pre-discharge mortality for EPIs or ELBWIs. The performance of the predictive model might be attenuated due to a limited number of these types of factors included in the predictive model. Additionally, this was a single-center study with a relatively small sample size, and the findings need further validation in multi-center, large-scale studies in the Chinese population.

## 5. Conclusions

Our predictive model, based on clinical characteristics and management strategies within the first day of life, effectively identifies mortality predictors/risk factors for EPIs or ELBWIs. Pulmonary surfactant use, Apgar-5, GA, and UVC use were negatively associated with pre-discharge mortality, while maximum appropriate FiO_2_ on the first day and multiplicity were positively associated. Our findings suggest that early intervention with surfactants and UVC is essential to improving survival rates in this population.

## Figures and Tables

**Table 1:** Characteristics of extremely preterm infants or extremely low birth weight infants on the first day of life ^1^.

Neonatal Factors	Surviving Infants (*n* = 119)			Deceased Infants (*n* = 92)			
	*n*	Median	P25, P75	*n*	Median	P25, P75	*p*-Value
Time observed, day	119	70	(61, 81)	92	2	(1, 9)	<0.001
Gestational age, week	119	27.7	(27.0, 28.4)	92	27.1	(26.1, 27.9)	<0.001
Mother’s age, year	119	31.5	±4.7	92	30.0	±4.7	0.037
Birth weight, kg	119	0.95	(0.90, 0.99)	92	0.90	(0.78, 0.98)	0.004
Apgar scores at 1 min	119	8	(5, 9)	92	6	(4, 8)	0.002
Apgar scores at 5 min	119	10	(9, 10)	92	9	(7, 10)	<0.001
Apgar scores at 10 min	119	10	(9, 10)	92	9	(9, 10)	0.043
Base excess, mmol/L	119	−6.80	(−9.35, −4.70)	92	−8.1	(−11.7, −5.5)	0.014
Blood lactate, mmol/L	119	2.9	(2.2, 4.7)	92	4.2	(3.1, 6.5)	<0.001
Blood pH	119	7.28	±0.11	92	7.24	±0.13	0.036
White blood cell count, 10^9^/L	119	8.42	(5.65, 13.88)	92	7.95	(5.60, 11.53)	0.279
Hemoglobin, g/L	119	156	(140, 167)	92	154	(144, 165)	0.869
Body temperature, °C	119	36.5	(36.2, 36.5)	92	36.2	(36.0, 36.5)	<0.001
Heart rate, BPM	119	144.2	±15.1	92	143.7	±18.7	0.713
Systolic pressure, mmHg	119	58	(51, 63)	92	55	(47, 65)	0.270
Diastolic pressure, mmHg	119	30	(25, 36)	92	29	(23, 37)	0.550
Average blood pressure, mmHg	119	38	(34, 43)	92	37	(32, 44)	0.584
Maximum appropriate FiO_2_, %	119	40	(30, 60)	92	60	(40, 100)	<0.001

^1^ *t*-test, Mann–Whitney U test, or Pearson’s chi^2^-test were used for continuous and categorical variables as appropriately. Abbreviations: FiO_2_, fraction of inspired oxygen.

**Table 2:** Crude hazard ratios of pre-discharge mortality for categorical variables.

Variables	Total (*n* = 211)	Deceased (*n* = 92)		Crude HRs	95% CIs	*p*-Value
		*n*	%			
Sex (Female vs. Male)	99	46	46.5	1.240	0.824, 1.866	0.302
Twins/multiplicity (Y/N)	45	27	60.0	1.891	1.206, 2.964	0.008
Cesarean (vs. Vaginal)	107	37	34.6	0.569	0.375, 0.863	0.007
UVC (vs. PICC)	100	34	34.0	0.534	0.350, 0.817	0.003
Antenatal steroids (Y/N)	156	60	38.5	0.563	0.366, 0.865	0.011
Ventilation (Y/N)	151	75	49.7	1.969	1.163, 3.335	0.007
Asphyxia at birth (Y/N)	106	56	52.8	1.840	1.210, 2.798	0.004
Small for gestational age (Y/N)	36	16	44.4	1.058	0.617, 1.815	0.838
C-reactive protein, mg/dL						0.928
<10	176	72	40.9	1.000	—	
≥10	8	3	37.5	0.949	0.299, 3.011	
Surfactants, dose						0.084
0	69	34	49.3	1.000	—	
1	64	31	48.4	0.969	0.595, 1.576	
2	52	20	38.5	0.688	0.396, 1.195	
≥3	26	7	26.9	0.421	0.186, 0.949	
Gestational age, week						<0.001
<26.0	23	18	78.3	1.000	—	
26.0–26.9	44	23	52.3	0.486	0.262, 0.901	
27.0–27.9	83	33	39.8	0.345	0.194, 0.614	
≥28.0	61	18	29.5	0.236	0.123, 0.455	
Birth weight, g						0.002
<600	18	14	77.8	1.000	—	
600–799	18	11	61.1	0.611	0.277, 1.348	
800–899	34	17	50.0	0.452	0.223, 0.920	
900–999	110	36	32.7	0.267	0.143, 0.496	
≥1000 g	31	14	45.2	0.390	0.186, 0.820	
Apgar score at 1 min.						0.009
0–3	35	23	65.7	1.000	—	
4–5	37	18	48.6	0.603	0.325, 1.119	
7–8	83	32	38.6	0.441	0.258, 0.754	
9–10	56	19	33.9	0.376	0.204, 0.691	
Apgar score at 5 min.						0.001
1–6	25	17	68.0	1.000	—	
7–8	49	27	55.1	0.593	0.323, 1.089	
9	54	23	42.6	0.459	0.245, 0.860	
10	83	25	30.1	0.289	0.156, 0.536	
Apgar score at 10 min.						0.131
3–8	39	19	48.7	1.000	—	
9	59	31	52.5	0.996	0.563, 1.764	
10	113	42	37.2	0.655	0.381, 1.126	
Base excess, mmol/L						0.015
<−10	64	37	57.8	1.000	—	
−10–−5.6	81	30	37.0	0.544	0.336, 0.882	
−5.7–−3.1	35	10	28.6	0.380	0.189, 0.764	
≥−3.0	31	15	48.4	0.690	0.379, 1.257	
Blood lactate, mmol/L						<0.001
≤1.6	26	4	15.4	1.000	—	
1.7–2.9	57	18	31.6	2.204	0.746, 6.514	
3.0–5.9	88	44	50.0	4.045	1.452, 11.263	
≥6.0	40	26	65.0	6.239	2.174, 17.902	
Blood pH						0.060
<7.2	56	31	55.4	1.000	—	
7.2–7.34	107	42	39.3	0.601	0.378, 0.956	
7.35–7.45	41	18	43.9	0.687	0.384, 1.228	
>7.45	7	1	14.3	0.186	0.025, 1.366	
White blood cell count, 10^9^/L						0.201
<5.0	38	17	44.7	1.000	—	
5.0–9.9	102	50	49.0	1.123	0.648, 1.948	
10.0–14.9	31	9	29.0	0.561	0.250, 1.258	
≥15.0	40	16	40.0	0.840	0.424, 1.663	
Hemoglobin, g/L						0.480
<145	63	24	38.1	1.000	—	
145–159	65	32	49.2	1.425	0.839, 2.419	
160–189	76	34	44.7	1.244	0.738, 2.097	
≥190	7	2	28.6	0.701	0.166, 2.968	
Body temperature, °C						0.002
<36.0	26	16		1.000	—	
36.1–36.4	96	49		0.782	0.445, 1.376	
36.5–37.3	89	27		0.384	0.207, 0.713	
Year of birth						0.014
2016 and before	39	24	61.5	1.000	—	
2017–2019	54	26	48.1	0.706	0.405, 1.231	
2020–2021	67	19	28.4	0.379	0.207, 0.693	
2022–2024	51	23	45.1	0.632	0.356, 1.120	

Abbreviations: PICC, peripherally inserted central catheters; UVC, umbilical venous catheterization.

**Table 3:** Hazard ratios of pre-discharge mortality for the predictors in the final stepwise Cox regression model ^1^.

Predictors	Total (*n* = 211)			Female (*n* = 99)			Male (*n* = 112)		
	HRs	95% CIs	*p*-Values	HRs	95% CIs	*p*-Values	HRs	95% CIs	*p*-Values
Surfactant, 1 dose	0.67	0.57–0.80	<0.001	0.63	0.45–0.89	0.009	0.68	0.55–0.84	<0.001
Apgar scores at 5 min, 1 point	0.83	0.75–0.92	<0.001	0.88	0.77–1.01	0.068	0.8	0.67–0.97	0.021
Maximum appropriate FiO_2_, 10%	1.17	1.08–1.26	<0.001	1.2	1.07–1.34	0.002	1.18	1.06–1.31	0.003
UVC (vs. PICC)	0.37	0.23–0.60	<0.001	0.30	0.15–0.61	<0.001	0.37	0.19–0.72	0.004
Twins/multiplicity (Yes vs. No)	2.03	1.21–3.39	0.007	2.38	1.09–5.20	0.030	2.05	0.98–4.29	0.057
Gestational age, week									
<26.0	1.00								
26.0–26.9	0.54	0.29–1.00	0.051	0.85	0.30–2.45	0.764	0.45	0.18–1.14	0.093
27.0–27.9	0.33	0.18–0.60	<0.001	0.38	0.17–0.83	0.016	0.29	0.11–0.76	0.012
≥28	0.29	0.14–0.59	<0.001	0.51	0.20–1.30	0.159	0.17	0.05–0.56	0.003

^1^ Predictors and their hazard ratios are derived from the stepwise Cox regression model based on the 13 features selected from the LASSO–Cox regression model (see Figure 1). *R*^2^: 0.334 (total), 0.353 (female), and 0.336 (male). FiO_2_, fraction of inspired oxygen; HRs, hazard ratios; PICC, peripherally inserted central catheter; UVC, umbilical vein catheterization.

## Data Availability

The data presented in this study are available on reasonable request from the corresponding author due to privacy and ethical restrictions.
